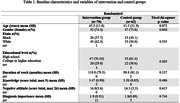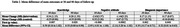# Randomized controlled feasibility trial to reduce stigma related to dementia among community health workers in Brazil: Preliminary results

**DOI:** 10.1002/alz.092205

**Published:** 2025-01-09

**Authors:** Carolina Godoy, Deborah Oliveira, Tassiane de Paula, Matheus Ghossain Barbosa, Vinicius Boaventura, Sara Evans‐Lacko, Cleusa P Ferri

**Affiliations:** ^1^ Universidade Federal de São Paulo, São Paulo Brazil; ^2^ Universidad Andrés Bello, Faculty of Nursing, Santiago Chile; ^3^ Millennium Institute for Care Research (MICARE), Santiago Chile; ^4^ Hospital Alemão Oswaldo Cruz, São Paulo Brazil; ^5^ Universidade Federal de São Paulo (UNIFESP), São Paulo, São Paulo/SP Brazil; ^6^ London School of Economics and Political Science, London United Kingdom; ^7^ Hospital Alemão Oswaldo Cruz, São Paulo, São Paulo Brazil

## Abstract

**Background:**

Dementia awareness and education are currently limited among healthcare workers and the general public, contributing towards the generation and propagation of stigma and discrimination against people with dementia worldwide. It is crucial to promote evidence‐based anti‐stigma interventions with a focus on stigma reduction.

**Method:**

This is a randomized and controlled feasibility trial of a group intervention aimed at improving knowledge and reducing stigma related to dementia among Community Health Workers (CHWs) (Trial Registration: RBR‐10xp637m). The Intervention Group (IG) (n = 70) received a 9‐hour intervention involving reflexive, educational, visual, and social contact activities related to dementia, and the Control Group (CG) (n = 62) was not intervened. Beyond sociodemographic characteristics, we collected data on dementia related stigma. For this preliminary analysis we will present data on: (1) negative attitudes (a composed score from 8 related question – the higher the score the more negative are the attitudes); (2) knowledge on dementia (a composed score from 5 related question – the higher the score the better the knowledge) and (3) a single question on the importance of dementia diagnosis disclosure (the higher the score the more important the participant think this is). The follow‐up questionnaires were reapplied 30 and 90 days after the intervention, using the same set of questions. We estimated and present the Mean Change (SD) in the IG and in the CG, the T‐test (p‐value), and the Mean Difference (95% CI).

**Result:**

There was no significant difference in any baseline measures between IG and CG. The preliminary results show that, compared to the CG, a significant increase in knowledge was achieved in the IG both at 30‐ (p = 0.007) and 90‐day follow up (p = 0.020), and a significant reduction in negative attitudes in both measure points (p<0.000 and p = 0.000 at 30‐ and 90‐day follow up, respectively).

**Conclusion:**

The preliminary findings indicate the intervention can lead to a sustained increase in knowledge and a shift in negative attitudes among CHWs over time. Further analysis are being carried out, including a qualitative assessment of the intervention, which will help improve the intervention to be applied in a randomized‐ controlled trial in the future.